# Plant-Associated Microbiomes: Crosstalk and Engineering to Improve Nutrient Use Efficiency (NUE) in Crops of Global Importance

**DOI:** 10.3390/plants15081265

**Published:** 2026-04-20

**Authors:** Pragya Tiwari, Kyeung-Il Park

**Affiliations:** Department of Horticulture and Life Science, Yeungnam University, Gyeongsan 38541, Republic of Korea; pki0217@yu.ac.kr

**Keywords:** agricultural crops, climate change, integrated nutrient management (INM), nutrient acquisition, nutrient transporter genes, plant–microbiome engineering

## Abstract

Global climate change is rapid and poses an alarming threat to agricultural production, significantly impacting economies. Modern agriculture has strongly emphasized improving nutrient availability in crops to address rising malnutrition and contribute to global food security. However, abiotic stresses, including warmer temperatures, drought, waterlogging stress, and elevated CO_2_, have critical direct and indirect effects on nutrient availability in plants. This systematic review was conducted in accordance with the PRISMA guidelines. The literature survey followed a time period of 2–5 months, during which the conceptualization, analysis, writing, and editing of the article were conducted. In the present era, it is essential to adopt measures to improve the nutritional value [enhance Nutrient Use Efficiency (NUE)] and nutrient management of plant-based foods. Plant-associated microbiomes have co-evolved with their plant counterparts and perform essential functions in nutrient acquisition, including microbial sensing and cross-talk with the plant host, nutrient uptake and sharing, and signaling mechanisms. In natural and agricultural ecosystems, plant microbiomes offer major opportunities and can be harnessed to sustainably supply essential plant nutrients and improve NUE in crops of global importance. Crop-associated microbiomes can be precisely tailored to achieve targeted outcomes, enhancing nutrient acquisition and utilization via microbiome engineering. However, bridging knowledge gaps, understanding microbial colonization, plant–microbiome dynamics, and adopting precise editing approaches are crucial to boost sustainable outcomes and crop productivity. By elucidating plant microbiome crosstalk and microbe–microbe signaling, a better understanding of microbe-mediated nutrient acquisition in plants can be achieved, defining key implications in global food security.

## 1. Introduction

### 1.1. Impact of Climate Change on Global Agriculture

In recent times, global agricultural production has been a driving force in supporting growing populations and impacting economies, shaped by diverse climates, soil types, and adopted practices. The staple crops like *Triticum aestivum* (wheat), *Zea mays* (maize), and *Oryza sativa* (rice) dominate the global crop production, while other specialized crops, including *Hodeum vulgare* (barley)*, Solanum tuberosum* (potato), *Manihot esculenta* (cassava), *Saccharum officinarum* (sugarcane), Canola, and others, are grown in different countries based on the environmental conditions (https://worldostats.com/country-stats/top-crops-produced-by-country/, accessed on 15 February 2026). The United States is a major producer of *T. aestivum*, *Z. mays,* and *Glycine max*, comprising crops for global exports as well as domestic consumption. Canada, with a colder climate, produces *H. vulgare*, *T. aestivum,* and Canola (oilseed crop) as key food sources and exports. In the Asian subcontinent, key agricultural crops include *O. sativa*, *T. aestivum*, and *S. officinarum*, which constitute major food sources for a large population. The countries, including Canada, India, and the United States, with variable climate conditions, are leading producers of *T. aestivum*, while *O. sativa* (a staple food crop) is predominantly grown in Asia. Although substantial efforts to promote food production and its availability have achieved considerable success, rising malnutrition and micronutrient deficiencies are alarming, particularly in low-income countries [1,2,3].

The global agricultural productivity is greatly impacted by climate uncertainties, affecting plant nutrition and quality, and changes in nutrient acquisition affect human nutrition [4,5]. The combined temperature of the ocean and land has warmed by 0.06 °C since 1850, posing a major challenge [6]. The indirect impact of climate fluctuations on agriculture is defined as: extreme climate conditions leading to drought and hailstorms, waterlogging and heatwaves; loss of biodiversity and soil fertility [5,7,8], and increased risk of pathogens and pests’ infection in crops at higher latitudes [9]. Moreover, studies have shown that crop growth periods have shortened due to a warmer climate [10], potentially leading to nutrient deficiencies. In crop plants, deviations from normal climate have a profound impact on protein and mineral accumulation, which is largely influenced by developmental stage, plant genotype, and stress duration [11]. Furthermore, any climate deviation alters interactions among crops, weeds, and pathogens, increasing water scarcity and ozone concentrations, and causing ecological disturbances. Climate change impacts plants to varying degrees, affecting developmental processes, functions, physiological responses, and morphological traits. While elevated CO_2_ (eCO_2_) promotes plant growth and productivity by increasing photosynthesis and water-use efficiency [12,13], grain quality is deteriorated, indicating that eCO_2_ disrupts carbon metabolism and mineral and nutrient uptake and use efficiency in plants (NUE) [14]. Subsequently, significant impacts of climate change on agricultural crops include reduced crop yields due to higher temperatures, increased evapotranspiration, water scarcity, and soil dryness (e.g., studied in crops—*O. sativa*, *T. aestivum*, *G. max*, and *Z. mays*) [15]. Crop quality: The nutritional value of rice in Japan and China under climate change was studied by Zhu et al. [16]. The study revealed that elevated levels of CO_2_ in the atmosphere caused a decrease in zinc (Zn), iron (Fe), protein, and vitamin B (B_1_, B_2_, B_5_, and B_9_) in rice, suggesting that variation in vitamin content poses a health risk to millions of rice consumers worldwide [16]. The climate fluctuations can have a profound impact on the nutritional content of staple food crops, specifically for populations that rely on plant-based food.

### 1.2. Key Impact on Climate-Driven Yield Variability

An increased shift towards plant-based diets is projected to increase the production of cereal crops to meet global food demand. Recent studies suggest an increase in global food demand by 30–62% by 2050 [17]. Worldwide, in regions with low production rates [18], sustainable intensification is a feasible approach to meet rising food requirements, increase production through higher yields, better cultivars, and bridge gaps in crop management [19]. These efforts for increased agricultural production in many regions are challenged by climate uncertainties, intensifying stress conditions, and variability in crop yield [20]. Under climate change, water availability, temperature changes, and CO_2_ concentration are the key factors that influence crop yield variability [21]. Discussing the individual drivers affecting crop yield during climate change, in C_3_ crops, photosynthetic rates are more affected by CO_2_ concentration than in C_4_ crops [22]. For e.g., in sorghum and maize, a higher crop yield can be achieved (up to 41%) under drought conditions, due to the effect of eCO_2_ influence on water retention and stomatal conductance. Studies based on free-air CO_2_ enrichment experiments showed that crop yield (rice and wheat) increased by approx. 17% under the eCO_2_ (550–590 ppm) when the experiment was carried out [23]. While in the presence of co-occurring drivers, the impact of climate change on crop yield may have more detrimental effects. Studies have shown that the combined effect of drought and heat stress has a greater adverse effect than the sum of individual stress in *H. vulgare* (barley) [24] and *Nicotiana tobacum* (tobacco) [25]. Under high temperature and drought, the grain growth and starch accumulation in two barley cultivars (Franklin and Schooner) were studied [24]. The results showed that drought conditions reduced grain weight (ca 20%) more than high temperature (ca 5%) in both barley cultivars, while the Franklin cultivar was more sensitive to higher temperature. In the Franklin cultivar, the weight of mature grain was reduced (under high temperature) due to a decline in duration and the rate of grain growth (8–12%). These studies highlighted that the combined stressors (drought and heat) cause a major reduction in grain weight under field conditions in key crops like barley, under climate change [24].

The interaction between higher temperatures, CO_2_ fertilization, variation in water availability, and impact on yield is exclusive to the cultivar, production system, and region of cultivation [26,27]. The knowledge of the climate change effect on yield variability and yield is crucial and would facilitate targeted measures (including improved breeding, insurance solutions, etc.) to cope with the uncertain conditions [28]. Webber et al. [29] used an assembly of crop models to quantify the effect of climate change on crop yield variation in European cropping systems (1984–2009), climate change impact to 2050. The results suggested that yield losses due to drought will increase for maize, while both maize and wheat will experience losses (due to drought) in years with low crop yield. The study further analyzed the effect of higher temperatures and elevated CO_2_: in maize, eCO_2_ improved the efficiency of transpiration, while in wheat, leaf area expansion and increased photosynthesis were observed. In the crops, efficient transpiration increased crop temperatures, which may further enhance heat stress. Moreover, climate change would cause yield gains for winter wheat and yield losses for grain maize, for the genotypes and blend of rainfed and irrigated production. It was also suggested that for both crops, drought stress caused key losses, and no increase in crop yield was observed with eCO_2_ [29].

Wang et al. [27] evaluated the effect of eCO_2_ on grain quality and yield in five cultivars of wheat. In the study, five Chinese wheat cultivars were cultivated under e[CO_2_] (800 ppm) and ambient CO_2_ (α[CO_2_], 400 ppm). Elevated CO_2_ promoted water-use efficiency and photosynthesis but depressed stomatal conductance. Moreover, eCO_2_ decreased N and increased Carbon (C) concentration, demonstrating cultivar-specific effects on the grain yield. The wheat cultivar, 02-1Shiluan, showed the highest grain yield (83.75  ± 3.73 g pot^−1^), grain number (2054 ± 90 a pot^−1)^, and shoot dry weight (215.13 ± 8.91 g pot^−1^), but the lowest thousand grain weight (42.16 ± 0.85 e.g.), respectively. Moreover, the analysis of N and C concentration in plants showed that the eCO_2_ decreases the concentration of N in the whole plant but increases C concentration in the straw and leaf, while the variation in N content differs among cultivars [27]. The study provided key insights into the effect of eCO_2_ on grain yield, quality, and rate of gas exchange in five cultivars of wheat.

### 1.3. Globally Important Crops and NUE: A Critical Parameter in Crop Productivity

In an era of a growing global population and rising food demand, adopting sustainable practices to achieve global food security is imperative [30,31,32]. For the evaluation of crop production, NUE is a critical parameter and is calculated as follows: the biological production based on per unit of applied nutrient and partial factor productivity (PFP). In cropping systems, these parameters facilitate the assessment of NUE and optimization of crop yield [33]. Among nutrients, the NUE estimate varies as follows: 15–20% (phosphorus), 30–50% (nitrogen), 70–80% (potassium), 8–10% (sulphur), and 1–2% for micronutrients (Fe, Zn, Cu, Mn, and B) [34]. Gerloff and Gabelman [35] introduced the term nutrient efficiency ratio (NER) to differentiate between nutrient-efficient genotypes and inefficient plant genotypes. The NUE is affected by multiple factors [36], including nutrient uptake influenced by nutrient influx kinetics, radial transport in roots, rate of influx, and nutrient availability or application in soil. On a global scale, NUE remains low across major crops, underscoring the need to devise strategies to improve NUE and support sustainable approaches. Globally, NUE and crop yields vary in different locations. Crop yields in South America, the United States, Western Europe, and China are high, surpassing 1000 kg N ha^−1^ yr^−1^, but yields in Africa, Central Europe, and Australia are less than 200 kg N ha^−1^ yr^−1^. NUE exhibits a varied pattern, with efficiencies that are typically around 50% in Asia and Australia but high (over 60%) in South America, the Central United States, Europe, and Africa. These variations result from cross-spatial interactions between agricultural management, climate, and soil health [36].

A global database on cropland nutrient budgets provides comprehensive information on nutrient use at different locations, variations in NUE, and a focus on the requirement for context-specific approaches to promote NUE [37]. Liu et al. [33] compiled a global database (covering data from 205 countries) and examined PUE and Nitrogen Use Efficiency for key crops between 1961 and 2018. The analysis revealed that PUE and Nitrogen Use Efficiency varied across crops and geographical locations (e.g., while *T. aestivum* showed optimal PUE and Nitrogen Use Efficiency in temperate zones, *O. sativa* demonstrated the best parameters in tropical regions). However, *Z. mays* demonstrated prominent nutrient inefficiencies and excessive Nitrogen (N) and Phosphorus (P), particularly in the United States and China. The study investigated the global-scale prevalence of low NUE in crops and provides a platform to guide region-specific initiatives to promote NUE and minimize dependence on fertilizers [33]. On a global level, Nitrogen Use Efficiency in cereal crops, including *Z. mays*, *T. aestivum*, and *O. sativa*, is approximately 33%, while the remaining 67% (in the form of N fertilizers) is lost due to surface runoffs, leaching, and soil denitrification [38]. As per recent statistics, Nitrogen Use Efficiency for cereal crops has improved from 31% in 2002 to 41% in 2015, attributed to crop management and improved cultivars. Omara et al. [39] discussed the trends in Nitrogen Use Efficiency in cereal crops worldwide, and estimated it as globally (35%), China (30%), the United States (41%), and India (21%), suggesting that fertilizer management practices could improve Nitrogen Use Efficiency. Ibn et al. [40] highlighted an integrated approach comprising physiology, agronomy, genomics, and genetics regulation, with potential to improve Nitrogen Use Efficiency in cereal crops. Beyond traditional methods, recent initiatives are focused on finding a feasible solution, including harnessing plant microbiomes and their functional implications in promoting crop productivity.

Plant-associated microbiomes hold significant potential to address current agricultural challenges and achieve higher crop productivity [41,42]. The plant-microbial symbiosis, particularly arbuscular mycorrhizal fungi (AMF) and fungal endophytes [43,44], is a key players that confer fitness advantages, including plant-growth-promoting (PGP) traits, nutrient acquisition, and biotic/abiotic stress tolerance, defining sustainable and eco-friendly solutions [45,46,47]. Camargo et al. [48] discussed the impact of plant microbiomes on nutrient availability in the Brazilian grassland ecosystem. The metagenome analysis revealed that plant-associated root microbiomes are rich in genes for N and P turnover and uptake of organic substances. In addition, the microbiomes of *Bryobacteraceae* and *Xanthobacteraceae* (bacterial families) demonstrated potential for P solubilization and transport, and nitrogen-cycle-related genes and nitrogen-fixing potential in *Isosphaeraceae*. The study was significant in showing that plant-associated microbes include a genetic repertoire to improve nutrient availability and novel nutrient turnover mechanisms [48]. On a theoretical level, plant-associated microbiomes can be employed to promote NUE via understanding the functional crosstalk between the plant host and the microbial counterparts, microbial sensing, signaling, and nutrient-sharing mechanisms. Addressing knowledge gaps in the complex molecular and physiological mechanisms in microbe-mediated nutrient acquisition would provide new directions in microbiome-assisted plant NUE research. While an empirical viewpoint suggests that harnessing the beneficial impact of the microbiome on plant health and productivity would enhance NUE [49], transforming agricultural production. Plant–microbiome engineering has emerged as a powerful tool to generate crops with increased stress tolerance and crop yield [50]. In globally important crops, understanding plant–microbiome crosstalks and finely tuning microbial interactions defines a prospective approach to improve plant health, nutrient cycling, and higher crop yields [51,52]. Multi-omics datasets can unravel specific gene-gene interactions in plant-microbial interactions and would facilitate stress tolerance, enhance nutrient efficiency, and promote crop productivity. While microbiome-based studies define immense possibilities, translating bench-to-field applications and addressing challenges requires a holistic understanding of the co-evolution of plant-associated microbiomes, sensing, and signaling mechanisms.

This article investigates the potential of plant-associated microbiomes in promoting NUE and how the complex molecular mechanisms shape microbial-assisted nutrient uptake in plants. However, limited knowledge restricts progress and necessitates further investigations. By elucidating plant microbiome crosstalk and microbe–microbe signaling in nutrient acquisition, a better understanding of microbe-mediated nutrient acquisition by plants under abiotic stress can be achieved, with key implications for global food security.

## 2. Materials and Methods

### Research Methodology: Bibliographic Source, Compilation, and Analysis

**Figure 1:**
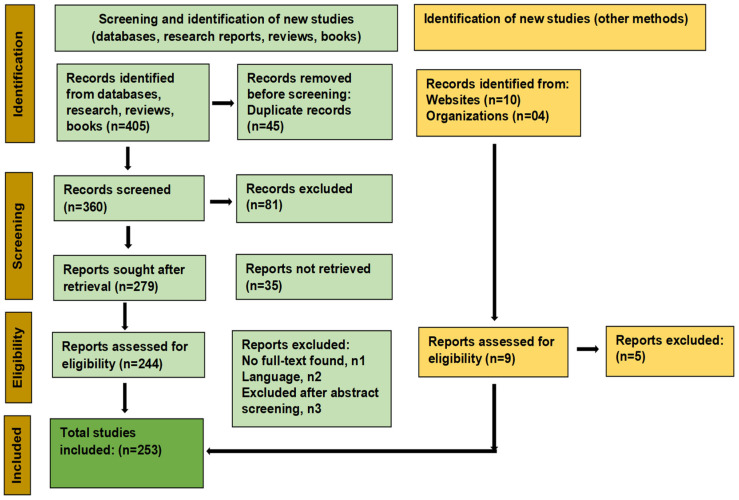
Study Flow Diagram.

The literature review was conducted according to the Preferred Reporting Items for Systematic Reviews and Meta-Analysis (PRISMA) guidelines [53]. Scholarly databases, including Google Scholar [54], Pubmed [55], and Scopus [56], were searched for studies related to the global scenario in climate changes and its impact on plant nutritional value, traditional and recent advances in NUE enhancement in plants (fertilizer management, plant biostimulants, and genetic modification) and an emphasis on how understanding plant microbiome crosstalk and engineering offer significant prospects in addressing NUE in the current decade. A literature search was carried out as per the following items: “global crops”, “climate change”, “agriculture”, “Nutrient Use Efficiency”, “plant microbiomes”, and/or “plant–microbiome engineering”. The literature was screened and compiled based on the following criteria: (a) peer-reviewed articles (original research, reviews, books, databases, conference abstracts, editorials, preprints) discussing concepts in plant NUE and its significance, (b) climate change and its impact on global crops and agricultural productivity, and NUE baselines, mechanisms of nutrient homeostasis in plants and traditional and modern approaches to enhance NUE; (c) Studies on deciphering plant–microbiome crosstalks and molecular mechanisms in nutrient acquisition and how advances in microbiome engineering can be harnessed to achieve a higher NUE in times of climate uncertainties. In addition, foreign and local books, organizations, and websites were consulted to obtain the relevant information. In total, (n = 405) records were identified, and duplicate records were removed (n = 45). The relevant literature was screened (n = 360), and irrelevant records were excluded (n = 81) (articles in which the outcomes were not of interest). Reports were sought after retrieval (n = 279), while some reports were not retrieved (n = 35). The exclusion criteria comprised: n1, articles with no full text available; n2, articles in other languages (non-English); and n3, articles excluded after abstract screening. To summarize, 253 studies were referred to compile and execute the literature review (Figure 1, Study Flow Diagram, according to PRISMA 2020 statement).

## 3. Results

### Nutrient Homeostasis in Plants: Regulatory Mechanisms to Maintain Nutrient Balance and Uptake

Modern agriculture faces multiple challenges, including a growing population, adverse environmental impacts of agricultural practices, high costs, and climate fluctuations. The extensive use of land for agriculture depletes soil nutrients, reduces plant yields, and causes low nutrient levels [57]. For optimal growth and development, essential minerals are required by the plants: including oxygen, carbon, and hydrogen (acquired from water and air), N, P and K (macronutrients) and nickel (Ni), copper (Cu), iron (Fe), molybdenum (Mo), zinc (Zn), boron (B), magnesium (Mg), calcium (Ca), chlorine (Cl) and sulphur (S) (micronutrients) [58]. The quality of plant-based food is affected by nutrient content, and it can be improved through biofortification (particularly for Zn and Fe), which are key to human well-being [59,60]. For the development of plants with high nutritional value, both factors, including nutrient availability and its efficient utilization by the plants, are of critical importance. NUE is the productivity estimate (nutrient uptake or loss per unit) that defines the capacity of plants to effectively acquire and utilize the available nutrients for biomass production [57], and includes nutrient storage, uptake, transport, and mobilization [61]. In globally important crops, NUE improvement has key benefits: efficient nutrient uptake, higher crop yield, improved soil fertility, greater tolerance to climate fluctuations, and higher returns on fertilizer inputs (https://www.cropnutrition.com/nutrient-management/understanding-nutrient-use-efficiency/ accessed on 20 February 2026).

Plants have intrinsic mechanisms for nutrient uptake, utilization, and translocation, which are modulated by environmental or developmental factors based on the plant’s requirements and nutrient availability [62]. In response to nutritional deficiency, signal transduction takes place in vascular tissue. Studies have demonstrated that vascular tissue (xylem and phloem) plays essential roles in growth and development by transporting signaling and nutritional molecules between plant tissues [62,63]. In plants, xylem tissue is actively involved in long-distance signaling and translocation of multiple signaling molecules, peptides, and hormones [64], while passively assisting the upward movement of nutrients and water. On the other hand, phloem plays a key role in transporting molecules, supporting long-distance communication, plant stress responses, and development [65]. Nutrient homeostasis in living cells is critical for maintaining balance and enabling biochemical processes to occur in a defined manner, which is fundamental to plant development [66].

The acquisition of multiple minerals in plants involves long-distance signaling networks and is precisely regulated by the plant vascular system. Nitrogen is critical for plant development and is taken up in nitrate (NO_3_^−^) form. Under low-nitrogen conditions, plants transport nutrients from shoots to roots, with vascular tissues facilitating nitrogen absorption and root growth [67]. In plants, N perception is governed by two pathways: the -trans-Zeatin-(*tZ*) pathway and the C-terminally encoded peptides-*CEP*-related pathway. When sufficient N is available, the *tZ* pathway regulates N signaling, while during N depletion, the *CEP* pathway governs N signaling in plants [68]. Another essential element for plant growth and development is phosphate, which affects crop yield [69]. In low phosphate conditions, plants either store phosphate or improve P uptake from the soil [69,70]. Moreover, two key signaling pathways regulate P in soil. The local signaling pathway in plants modifies root system architecture and increases Pi acquisition [71]. The local and systemic signaling pathway jointly governs how plants respond to P availability in the soil [71]. The systemic response in plants detects P concentration, improves uptake, and maintains P homeostasis via recycling [72,73]. Furthermore, P acquisition and utilization in plants are coordinated via crosstalk between local and systemic signaling pathways [74,75]. Studies have shown that phosphate starvation-induced (*PSi*) genes are upregulated when plant sucrose levels increase, under low phosphate conditions [76]. In addition, plant hormones and transcription factors act as signaling molecules under phosphate depletion [59]. Regulatory functions in remodeling of the root system (low phosphate concentration) are performed by hormonal crosstalk (ethylene, gibberellin, auxin, strigolactones, and ROS-signaling pathways) [77,78].

Iron (Fe) is another key mineral that is enriched in soil through multiple biogeochemical processes, including microbial activity, mineral weathering, and the decomposition of organic matter. In soil, Fe is present in soluble and insoluble forms- the soluble form of Fe is readily available to the plants, and iron oxide undergoes sequestration and is long-term retained in the soil. Grillet et al. [79] discovered a C-terminal consensus designated as IRON MAN (*IMA*), a highly conserved peptide sequence (found in angiosperms) with a key role in Fe uptake in plants [79]. Overexpression of *IMA* in plants caused increased iron uptake in roots and overall iron accumulation, improving plant tolerance to Fe deficiency. Kobayashi et al. [80] demonstrated that the absence of *IMA* causes iron deficiency, and it performs critical functions in Fe homeostasis. Moreover, studies by Zhai et al. [81] elucidated the function of phloem-specific iron transporters in Fe transport, validating the key role of phloem tissue in Fe distribution and homeostasis in plants. Plants have inherent mechanisms to maintain nutrient homeostasis; however, this balance may be affected by changing climate conditions, impacting crop productivity. Advanced approaches are required to address the impact of climate change on NUE and create cultivars with efficient nutrient acquisition and utilization efficiencies.

## 4. Discussion

### 4.1. Traditional and Modern Approaches to Promote Nutrient Use Efficiency (NUE) in Plants

**Figure 2:**
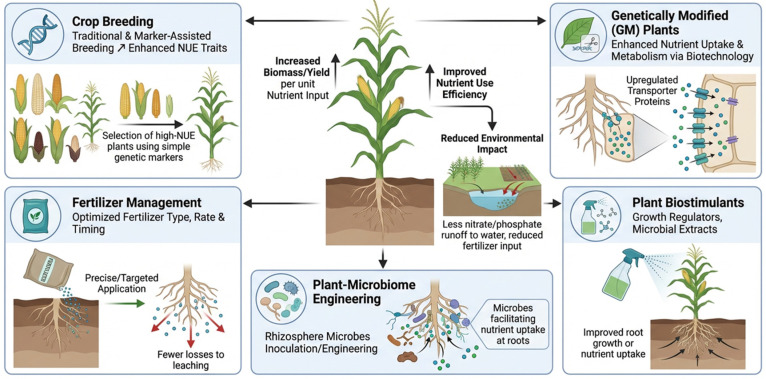
Key approaches to enhance nutrient use efficiency (NUE) in crops include crop breeding, fertilizer management, genetically modified plants, and plant–microbiome engineering (source: created with FigureLabs for scientific illustrations). Traditional and modern approaches have been significant in enhancing nutrient acquisition and utilization and/or creating improved plant cultivars with higher assimilation and nutrient uptake, improving crop yields.

In plants, the efficient utilization of nutrients is crucial for crop productivity and necessitates the development of sustainable approaches [82]. The presence of low nutrients or inefficient utilization by plants can decrease soil fertility, increase the emission of greenhouse gases, and lead to water contamination (due to leaching of nitrates). Several factors affect nutrient utilization by crops, including crop type, soil properties, cropping systems and management, and fertilizer use, and it is important to understand them in the context of NUE. Traditional and modern approaches have substantially contributed to improving plant NUE and comprise biostimulants, agronomic approaches integrated with advanced genetics, and nutrient management. Moreover, advances in biotechnology and genetics (including genome editing and plant breeding) have produced improved plant varieties with better N assimilation and uptake. In addition, microbial inoculants (mycorrhizal fungi and nitrogen-fixing bacteria) have reduced fertilizer usage and promoted N availability in plants. Figure 2, Key approaches to enhance nutrient use efficiency (NUE) in crops include crop breeding, fertilizer management, genetically-modified plants, and plant–microbiome engineering.

In the agricultural sector, the development of nutrient-efficient crops is significant for transitioning towards a bio-based economy and achieving sustainable food production [83,84]. While N and P are critical nutrients, parameters, including environmental factors, resources (e.g., water), and micronutrients, are essential for plant development [85]. The current biotechnological approaches prioritize nutrient transporters, root exudate secretion, root modifications, and nodule-forming microbes (rhizobial bacteria and arbuscular mycorrhizal fungi (AMF). The NUE initiatives are centered around increasing nutrient remobilization and translocation, the release of stored nutrients, and improving metabolic efficiency [34,86].

Sustainable crop production through NUE management requires a multi-dimensional strategy combining technological advances, agronomic approaches, and government support. While efficient nutrient utilization is the primary goal in sustainable farming practices [1], a rapidly fluctuating climate has complicated nutrient management due to warmer conditions, climate fluctuations, and changes in precipitation that affect fertilizer efficacy and crop nutrient uptake [87]. In plants, effective nutrient management is crucial to enhancing crop yields and productivity and creating resilient agricultural systems. Integrated nutrient management (INM) has been introduced to promote the uptake and utilization of available nutrients in vegetables and other crops [88]. INM has been significant in improving physiological and biochemical attributes and plant yield, including *S. tuberosum* (potato), *Allium cepa* (onion), *Z. mays* (maize), *Solanum lycopersicum* (tomato), and *Vigna unguiculata* (cowpea). INM has been pivotal in the growth of multiple field crops and vegetables, enhancing nutrient acquisition, plant growth, and characteristics, and significantly improving plant yields [89,90], highlighting novel possibilities.

### 4.2. Fertilizer Management Approaches

A changing climate severely affects crop yields and calls for soil fertility management through judicious use of fertilizers [91,92]. Fertilizers (mostly containing N, P, and K) are widely used to improve agricultural yields and productivity; however, these have certain disadvantages. According to the Food and Agriculture Organization (FAO), approximately 45 Mt of phosphorus (P), 38 Mt of potassium (K), and 109 Mt of nitrogen (N) were used in agriculture in 2017, highlighting excessive application and adverse impact on the environment.

To optimize fertilizer use, it is important to understand plant nutrient requirements and implement management approaches. Shohag et al. [93] studied the combined impact of oxygen and phosphorus fertilization on plant pod yield, growth, and phosphorus acquisition in *Phaseolus vulgaris* L. The results showed that the co-application of superphosphate and calcium peroxide promoted pod production and vegetative growth [93], suggesting that hypoxic stress in plants can be mitigated by the application of oxygen-based fertilizers, while promoting PUE and plant productivity. Yu et al. [94] examined the effect of microbial P mobilization (in soil) and P uptake in *O. sativa* via *Bacillus megaterium* and husk biochar application. The results demonstrate a profound improvement in available P (in soil) and a favorable impact on bacterial population, increasing P availability. Wang et al. [95] determined N application rates and irrigation levels on different plant parameters (nitrogen use efficiency, soil nitrate-N (NO_3_^−^-N) residues, and sugar yield) in sugar beet cultivation. In the study, an increase in N uptake and sugar yield was observed; however, nitrogen use efficiency declined. The study provides key findings to promote N and water management in sugar beet cultivation across the globe.

Zou et al. [96] examined the global trends of P use in cropland (in different countries) and presented a P-budget database (input and output of the crop production) for each country and type of crops between 1961 and 2019. The study emphasized the need to increase PUE in crop production to 68–81% to address P management bottlenecks. Chatzistathis et al. [97] and Jiang [98] discussed that in some wheat cultivars, Fe and Mn efficiency was related to efficient nutrient utilization and not due to higher Fe and Mn accumulation. Other studies have documented major differences in nutrient utilization efficacy by various plant species, affected by factors including genetic traits (better nutrient utilization capacities), root capacity for secretion of exudates (which promotes nutrient uptake), and root mycorrhizal colonization [97]. In most agricultural systems, NUE is limited by the limited efficiency of many cultivars to acquire and utilize available N and higher losses of N fertilizers, limiting nutrient availability [99,100].

### 4.3. Plant Biostimulants and Biofertilizers: Sustainable Solutions to Enhance NUE

Recently, there has been growing interest in eco-friendly, bio-based methods to boost agricultural production and mitigate the adverse impacts of climate change. According to The European Biostimulant Industry Council (EBIC), biostimulants are defined as follows: “Plant biostimulants contain substance(s) and/or microorganisms whose function is to be applied to plants or the rhizosphere is to stimulate natural processes to enhance/benefit nutrient uptake, nutrient efficiency, tolerance to abiotic stress, and crop quality” (reported in the European regulation n. 2009/2019) (European Biostimulants Industry Council, https://biostimulants.eu/) [101]. The biostimulants are microbial and non-microbial biostimulants, based on the active constituents. The non-microbial stimulants include humic substances, seaweed and plant extracts, plant growth regulators, and protein hydrolysates, while the microbial biostimulants contain chemicals (e.g., melatonin), biostimulants from other sources, and phytohormones. In addition, substances such as chitin, chitosan oligosaccharides, and natural polymers can be used as biostimulants [88]. Microbial biostimulants are increasingly investigated as sustainable solutions, and representative examples include: plant growth-promoting rhizobacteria (PGPR), non-mycorrhizal and mycorrhizal fungi, and *Trichoderma* spp. [102].

These biostimulants comprise different types of microorganisms and other substances that promote plant growth, nutrient acquisition, flowering, development of fruits, biotic/abiotic stress tolerance, and crop productivity [103]. Moreover, these are applied in very low concentrations and increase protein content (in grains), shelf-life, and nutritional quality of crops [104]. In the present decade, biostimulants have increased NUE, attributed to nutrient availability and better root development [105]. The direct mechanisms include higher expression of nutrient transporter genes (particularly P- and N-related) to fulfill plant requirements. The representative studies are as follows: application of microalgae (*Chlorella vulgaris* and *Messastrum gracile*) in *Cattleya labiata* promotes in vitro propagation [106], seaweed-based extract in *Cucumis sativus* improves fruit yield and growth [107], arbuscular mycorrhizal fungi in *Olea europaea* L. promote rooting and quality of seedling [108], microbial metabolites in *Pyrus communis* L. increase auxin production and rooting [109], including other examples. Amirkhani et al. [110] showed that plant seed coating with biostimulants improved plant parameters and N uptake compared to the control. Moreover, the application of biostimulants as seed coating in broccoli plant (10-day-old seedlings) showed improved shoot and root growth, compared to control plants [111]. The translational success of the biostimulants is based on the fact that they utilize all the available nutrients (N, P, and K, micro- and macronutrients), assist in nutrient cycle optimization, and minimize nutrient loss due to emissions, leaching, and runoff, including other factors.

Schutz et al. [112] discussed the application of microbial inoculants as a promising approach to promote plant yield, Nitrogen Use Efficiency, and PUE based on a meta-analysis of key studies. The key findings include: biofertilizers demonstrating P-solubilizing and N-fixing traits showed the highest potential to promote crop yield, while AMF (assist in P uptake) were most effective. Biofertilizer application increased PUE by 7.5 ± 0.8 kg yield per kg P, more visible in legumes, while on average, Nitrogen Use Efficiency improved by 5.8 ± 0.6 kg yield per kg N fertilizer, achieved through biofertilization [112]. Particularly, AMF associations are important in plant nutrient uptake, due to their ability for biological fertilization, nutrient (including N), and water uptake by plants. In addition, AMF fungi hyphae efficiently utilize inorganic N and enable plant access to N in the soil [113].

### 4.4. Genetically Modified Crops to Improve Crop Nutrient Efficiency

The expanding horizons of sustainable agriculture have increasingly recognized the contributions of genetically modified crops [114,115]. Furthermore, environmental and development modulation of multiple processes governing crop nutrient efficiency can be achieved via genetic engineering. While significant advances in improving plant traits have witnessed translational success, improving nutrient efficiency in plants through genetic modification has been gaining recognition recently [116,117]. Genetic modification that focuses on nutrient transport holds promise to improve nutrient acquisition efficiency (NAE) and reduce environmental impacts [118,119,120]. In the present decade, studies have largely investigated either soil conservation or plant nutrient uptake, with little knowledge of how genetic modification can precisely address multiple concerns [118,121]. It necessitates future research to understand genetic modification synergies in plant–soil systems, expanding horizons in creating high-value traits in crops [115].

For plant growth and development, nutrient uptake is essential and often occurs through the root system, particularly for micronutrients (N, P, and K) [122]. In key crops, namely wheat, barley, rice, soybean, maize, and rapeseed, 79 P-efficient and 248 N-efficient genotypes have been identified [123]. In addition, genes regulating PUE or Nitrogen Use Efficiency have been discovered, including transcription factors, signaling molecules, transporters, metabolic enzymes, and their networks [124,125,126]. The process of nutrient uptake is governed by multiple factors, including plant characteristics, namely physiology, genetic traits, and morphology, and environmental factors, namely available nutrients in soil, pH, and moisture, that work in a coordinated manner [127,128,129]. Genetic modifications of key crops provide novel insights and support conservation efforts- improved soil-binding properties by plant root architecture modifications [130]; plant tolerance to stress conditions, including strong winds, UV-B radiation, drought, and others [131,132,133], and enhanced plant nutrient uptake efficiency [134,135]. Wang et al. [123] discussed the integrated approaches and advances: how genetic modification aims to develop plant cultivars with improved PUE/Nitrogen Use Efficiency in economically important crops.

Sathee and coworkers [121] explored genome-editing targets to promote nutrient-stress adaptation and plant NUE in crops of socio-economic importance. The authors discussed the role of negative regulators of nutrient signaling that can be targeted to improve stress-mediated signaling and nutrient uptake in crops. Moreover, precise changes can be achieved (promoter engineering by CRISPR/dead, Cas9 (dCas9) cytosine and adenine base editing) [121]. Kumari et al. [136] discussed how the CRISPR/Cas technology for precise genome editing has created crops with high nutritional value. Moreover, genetic modifications in legumes and some grass species focused on developing deeper and larger root systems [137]. Studies have found that DEEPER ROOTING 1 (*DRO1*) governs grain yield and influences root systems in *O. sativa*, promotes tolerance to drought and salinity stress in *Prunus persica*, and *Arabidopsis* [138,139]. Yan et al. [140] performed CRISPR-based gene editing of the flavone pathway in *O. sativa*. The study showed that the genetic engineering improved biological N fixation and reduced fertilizer application in cereals. Wang et al. [141] adopted a transgenic approach to modify *OsMYB305* (MYB transcription factor) in rice. The genetic modification of the plant increased N absorption by redirecting carbohydrate flow toward cell wall biosynthesis. These crop engineering initiatives enhanced key traits related to nutrient acquisition and soil structure. Maurya et al. [142] used genome editing to precisely delete the W-box cis-element from the promoter of *OsPHO1;2* (phosphate transporter gene) in *O. sativa* and depressed gene expression. The genetically modified lines showed increased root-to-shoot phosphate translocation, improved grain yield, and P uptake. These genome-editing studies enabled Nitrogen Use Efficiency/PUE optimization and gene modification, opening new opportunities for enhanced nutrient utilization and crop productivity.

### 4.5. High-Nutrient-Use-Efficient Crops

In the present decade, the adoption of sustainable agricultural practices and a decreasing reliance on non-renewable fertilizers are necessary and can be achieved through improved PUE and Nitrogen Use Efficiency in major crops [123]. To generate crops with high NAE, genetic and molecular investigations have focused on identifying elite genotypes, overexpressing nutrient transporter genes, and enhancing nutrient utilization through noncoding regulatory RNAs and transcription factors [143]. Advanced biotechnological approaches have been instrumental in enhancing NUE in legume and cereal crops. Marker-assisted breeding (MAB) has been widely applied: e.g., representative studies include—*T. aestivum*: potassium use efficiency [144]; *Vigna radiata*: PUE [145], and *T. aestivum*: nitrogen-related traits [146,147]. Furthermore, biotechnological methods are preferred over breeding due to precise genetic manipulation, shorter experimental time, and the removal of the cross-genus barrier [148]. Studies on NUE improvement documented the functional characterization of multiple genes encoding sensors, metabolic enzymes, and nutrient transporters [148,149]. Recently, trait engineering targeting four traits (nutrient uptake, availability, translocation, and utilization) has been attempted to enhance PUE or NUE [125,126]; however, distinct environmental and biological challenges limit further evaluation. Schneider et al. [150] studied that blocking *ZmCIPK15* (a CBL-interacting serine/threonine-protein kinase that governs root gravitropic response) caused enhanced shoot biomass and N uptake under low N in fields. Oldroyd and Leyser [151] elucidated the molecular mechanisms in *A. thaliana*, demonstrating that efficient N uptake is determined by the root foraging under uneven N supply and that N-foraging traits are utilized under low N conditions. Araus et al. [152] developed an NUE regulatory network using an algorithm and transcriptomic data from microarray studies in *Arabidopsis*. The study found that the *AtBT2* (Bric-a-Brac/Tramtrack/Broad (*BTB*) gene family) is a key player, and its mutation improved Nitrogen Use Efficiency under low-nitrate conditions. Attempts to improve PUE in plants have yielded interesting results. Lu and coworkers [153] showed that genetically modified tobacco expressing the malate dehydrogenase gene caused biomass increase when aluminum (Al), Fe, or calcium-bound phosphorus was added, suggesting that modification of organic acid exudation can be a feasible approach. Furthermore, crop engineering targeting traits involved in mobilizing insoluble P in soil (e.g., root exudation of organic acids or siderophores) and improving P uptake [86] is a promising area for future research. In addition, integrated approaches, including speed breeding with mutagenesis, genomic selection, and pre-breeding, can be effective for developing high-nutrient-use-efficient crops.

## 5. Plant Microbiome Crosstalk and Engineering: Harnessing Plant Microbiomes for Sustainable NUE in Plants

In the present era, global agriculture is facing critical challenges, including climate fluctuations, depleting resources, and global food shortages, projecting the need for sustainable and eco-friendly alternatives [154]. The plant microbiome comprises microbial communities (and their genomes) and defines novel attributes to decipher these intricate interactions, promote agricultural output, minimize pesticide usage, and support a sustainable ecosystem [155,156,157,158]. Plant-associated microbiomes are closely linked to their plant hosts and colonize plant tissues (internal or surface association), comprising diverse organisms such as fungi, protists, nematodes, bacteria, and viruses [159,160]. These microbial communities perform different functions [161,162], including plant growth promotion, nutrient acquisition, and tolerance to stress conditions [45,46], while plants support microbial growth and adaptation, demonstrating mutualism. The plant microbiome is compartment-specific and comprises the endosphere (within plant organs above or below) [163], rhizosphere (soil surrounding plant roots) [164], and phyllosphere (above-ground plant parts) [165]. Xiong et al. [166] reported that the plant signaling system selectively recruits microbial species based on their deeper penetration into plant compartments. Recent biotechnological advances have been instrumental in providing key insights into plant–microbiome crosstalks and how these microbes govern plant physiology, fitness, and colonization, facilitated by genome-wide association studies (GWAS) [167]. In addition, multi-omics initiatives predicted the genes that promote microbial interaction and crosstalk [50]. Plant-microbiome interactions drive plant development and N acquisition, e.g., bacterial genus *Sphingopyxis* enhances lateral root development and alters auxin biosynthesis to promote N acquisition under stress conditions.

In the plant root-soil interface (rhizosphere), root exudates secrete organic nutrients, which facilitate the colonization of beneficial microbes (endophytes or epiphytes) that establish dynamic associations with plant roots. Li et al. [168] showed that the rhizosphere microbiomes govern plant fitness and performance. Particularly, the plant-associated fungi form a hyphal network that extends beyond the rhizosphere and promotes nutrient acquisition in the plant-microbial association. These interactions are governed by environmental conditions, microbiome composition, plant genotype, developmental stages, and microbial interactions [50]. Studies are increasingly investigating these parameters for improving NUE, presenting both opportunities and challenges. Recent scientific advances linking specific microbial taxa to microbe-mediated processes have benefited from genomic information (metagenomics, genomics, and marker genes) and taxonomic classification based on function [169,170]. The plant host provides a diverse environment and harbors distinct microbial species in their rhizosphere that, in turn, promote plant fitness and beneficial traits [171,172]. Plant root exudates are the primary means of communication between plants and their microbial counterparts, eliciting responses such as competition for resources, nutrient absorption, and attracting diverse microbes [173,174]. In addition, organic nutrients secreted from root exudate mediate microbe-mediated tolerance to abiotic stresses, including disease, drought, and nutrient deprivation [175,176,177,178,179]. Wang G et al. [180] discussed that during the growth of *Z. mays*, different types of root exudates enhanced PUE and plant yield by altering key microbial species present in the soil. In the study, the application of inositol, succinic acid, and luteolin considerably improved plant root biomass, and different exudates increased soil P availability by modifying soil fungal and bacterial communities [180].

Plant symbiosis with rhizobial bacteria and AMF defines efficient biological approaches (natural or engineered) to enhance plant nutrient uptake. In crops such as *T. aestivum*, *O. sativa*, and *Z. mays*, AMF associations assist in nutrient uptake and the acquisition of fixed carbon from the plant host. This symbiosis is significant because it enhances nutrient acquisition (particularly minerals such as Cu, N, P, and Zn) beyond the rhizosphere [181,182]. For example, P uptake rates in AMF-associated plants are higher than in non-AMF plants (in limited P conditions) [183], and studies suggested that in *O. sativa*, up to 70% of the acquired P is through microbial symbiosis [184]. Studies have demonstrated that phosphate translocation from fungi to host roots is regulated by polyphosphate and its breakdown, which releases arginine from root cells, resulting in N and phosphate flux to plant roots [185,186]. Sondergaard et al. [187] reported that proton transporters on fungal and plant membranes facilitate phosphate transport from fungi to the plant host. Studies showed that the overexpression of Hþ-ATPase *OsHA1* (from *O. sativa*) enhanced phosphate uptake and improved nutrient exchange in the arbuscular-plant association [188]. Tamura et al. [189] and Yang et al. [184] characterized *GmPT7/10/11* from soybean and *OsPT11/13* from rice (AM-induced phosphate transporters). The studies revealed the biochemical and molecular mechanisms underlying P uptake in plants, facilitated by the AMF association.

Symbiotic nitrogen fixation between legumes and diazotrophic bacteria (particularly the *Rhizobium* genus) promotes N input in nature, responsible for fixing 122 million tons of N annually and including nearly half of N in agricultural systems [190]. Lopez-Arredondo et al. [191] evaluated the effects of nitrogen-fixing bacteria on *T. aestivum*, *Z. mays*, and *C. arietinum* (chickpea) productivity and to reduce fertilizer usage. The study examined inoculation methods, crop-bacterial specificity, and field-microcosm compatibility. The results showed that *Rhizobium* promotes the yield of *T. aestivum*, *Azospirillum* promotes *T. aestivum* yield, and *Azotobacter* positively impacts *C. arietinum* growth, considerably improving crop productivity. Zhang et al. [192] demonstrated that different varieties of rice show variation in Nitrogen Use Efficiency, attributed to the higher nitrogen-cycle related bacteria and efficient N transformation in *O. indica* roots than *O. japonica* varieties. The symbiotic association and nutrient exchange are governed by the expression of symbiosis-induced nodule genes in legumes [193]. Li et al. [194] showed that *GmEXPB2* (a β-expansin gene) affects nodulation in *G. max* by regulating plant growth, symbiotic N_2_ fixation, and modifying root architecture. Subsequently, free phosphate released during symbiotic N_2_ fixation acts as a feedback loop to limit N_2_ fixation under excessive phosphate [195]. Qin and coworkers [196] showed that *GmPT5* (phosphate transporter gene) maintains phosphate homeostasis (under low phosphate conditions), alters N/P content in plants, and regulates nodulation. These studies provide a foundation for future research to improve N/P efficiency and crop productivity through nodulation in plant-microbial interactions.

### 5.1. Plant Microbiome Crosstalk in Nutrient Acquisition

#### 5.1.1. Nitrogen (N) Acquisition

In many ecosystems, N defines the limiting factor in plant growth, usually present in nitrate form (organic N forms and ammonium in small parts). Microbes display multiple mechanisms in N uptake and transport in plants, via processes including nitrification, N-fixation, mineralization, and transport. In leguminous plants, a symbiotic association between root nodules and symbiotic bacteria facilitates biological N-fixation, converting atmospheric N into ammonia via the nitrogenase enzyme. The fixed ammonia is taken up in the form of amino acids and transported to the shoots [197]. The non-nodulating diazotrophs *Pseudomonas stutzeri* and *Azospirillum brasilense* are present in the rhizosphere and fix atmospheric N via plant root exudates, improving nutrient availability [198]. The atmospheric nitrogen (N_2_) is fixed by diazotrophic bacteria, which actively transport nitrate (NO_3_^−^) and ammonium (NH_4_^+^) to the plant. Moreover, AMF convert arginine to urea and to NH_4_^+^ [199]_._ In addition, AMF promotes nutrient availability through mycelium-assisted long-distance transport and arbuscules, directly to the cytoplasm of the host and the mineralization process. The improved N uptake in plants is mediated by the expression of nitrate-transport-related and ammonium genes, induced by other microbial species [200]. Soil-inhabiting microbes sometimes act as biological nitrification inhibitors via competition and production of chemicals, thereby enhancing Nitrogen Use Efficiency [201].

#### 5.1.2. Phosphorus (P) Acquisition

Microbes assist in P acquisition for plants through multiple mechanisms: (a) organic P compounds mineralization (e.g., phosphomonoesters), and enzymatic mineralization (e.g., phosphatase) [202], and (b) organic acid exudation mediated solubilization of inorganic P. AMF associations display efficient mechanisms of P transport and uptake; P acquisition from soil is assisted by a Pi transporter (fungal induced phosphate transporter 1) and convert Pi into polyphosphate, it further transforms multiple times and acquired by the plant in exchange of lipids and sugars [199]. Furthermore, plant access to P is facilitated by AMF P uptake mechanisms [203] and non-symbiotic microbes, including *Mucor* spp., *Bacillus* spp., *Penicillium* spp., and *Pseudomonas* spp. [199]. These microbial species promote P uptake through P transporter gene upregulation and increase P availability, e.g., De Zutter et al. [204] stated that in plant roots, bacteria possessing multiple P-solubilizing traits are enriched and mitigate P stress under P-deficient conditions. In crops like rice, potato, and tomato, AMF signal upregulation of P transporter genes (LePT4), which causes increased P transport from soil to fungal mycelium and enhanced P uptake [49]. It is shown that P uptake is a crucial parameter for plant-AMF symbiosis, since AMF colonization of plant roots is decreased during suppression of P transporter genes [205]. To improve P acquisition, plants also recruit microbial communities (non-symbiotic) via multiple mechanisms: In maize, Actinobacteria in the rhizosphere mitigate P deficiency during the seedling growth and improve P acquisition [206,207]. These symbioses are bidirectional as well as influenced by interactions across kingdoms. An example shows that bacterial and AMF association is more effective in enhancing transporter gene expression than individual microbes, while AMF increase organic P mineralization through fulfilling bacterial nutrient requirements [208].

While most studies have emphasized the microbiome-mediated nutrient acquisition for N and P, recent investigations suggest that plant and soil microbiomes are significant in macronutrient (including Mg, S, and K) acquisition. Multiple species of fungi and bacteria catalyze K solubilization from minerals via processes, namely chelation, acidolysis, and others, and induce K transporter gene expression in plants [209,210]. Moreover, microbes-assisted uptake of micronutrients (Mn, Fe, and Zn) [211,212] has been documented. For example, *Bradyrhizobium* spp. and *Rhizobium* spp. produce different siderophores (citrate and rhizobactin) that can bind to micronutrients and assist in direct uptake. In another study, Fe solubility and acquisition were improved through plant-derived and microbial organic acids and Fe mobilization [212]. During Fe deficiencies, microbial volatile organic compounds (VOCs) activate plant response and initiate plant regulatory components for Fe uptake [213]. While some progress has been achieved in understanding how the plant-associated microbiome promotes nutrient acquisition, molecular mechanisms remain less understood. Future research necessitates the identification of molecular processes governing nutrient availability and microbial-assisted pathways to harness functional attributes of microbiomes in plant nutrient acquisition.

Plant nutrient acquisition, assisted by microbiomes, defines a complex process, and further research should integrate the environmental conditions impacting plant–microbiome interactions. Multiple aspects of plant-microbe and microbe–microbe crosstalk and their response to abiotic stress require an in-depth understanding. Although some success has been achieved, knowledge about genes that affect nutrient acquisition (nutrient transporters, induce transcriptional regulation), microbial traits, signaling molecules, and communication networks needs to be characterized [214]. Recent advances have highlighted the prospects of microbiome engineering; however, it requires precise evaluation so as not to impact plant productivity, native soil health, or the environment.

#### 5.1.3. Microbe–Microbe Signaling in Nutrient Acquisition

The signals produced by the microbes, plants, and themselves are perceived and interpreted by rhizosphere-inhabiting microbes. The plant-associated microbiome communicates with its host and surroundings and induces plant immunity, growth, stress tolerance, plant nutrition, and others [215]. The key signaling molecules include: diketopiperazines, N/acyl homoserine lactones (AHLAs), volatile organic compounds, diffusible signal factors, and phytohormone-like molecules [216,217,218,219]. The plant-microbial communication may occur at the individual or at the microbiome levels and is studied in fungi and soil bacteria, and recently in protists and nematodes [220,221].

The signaling takes place between microbial interspecies and intraspecies, through the quorum-sensing (QS) mechanism, comprising the synthesis and detection of autoinducers. The transcription of multiple QS-regulated genes (including chemotaxis, biofilm formation, and virulence factors) is activated or deactivated by the autoinducers. Within microbial communities, this process assists microorganisms in governing behavioral changes and cell density [178]. An example of a QS molecule, N-acyl homoserine lactones (AHL), acts on Gram-negative bacteria (e.g., *Burkholderia* spp., *Serratia* spp., and *Pseudomonas* spp.). Moreover, the intra- and interspecies communication is performed by peptides in Gram-positive bacteria [222]. The QS mechanisms are significant for interkingdom communication that occurs between plant-associated fungi and bacteria; however, little information about specific mechanisms is available [223,224]. Signaling in inter-kingdom and intra-kingdom is carried out by volatile organic and inorganic compounds (VICs and VOCs) at low concentration and long distance [225,226]. Other compounds, including trehalose, thiamine, oxalic acid, and others, function as signaling molecules [227]. E.g., *Laccaria bicolor* S238N (a mycorrhizal fungus) produces trehalose, which acts as a chemoattractant for *Pseudomonas fluorescens* BBc6R8, while thiamine produced by Mycorrhiza Helper Bacteria promotes the growth of mycorrhizal fungi. The mechanisms in microbe–microbe signaling facilitate microbes to survive in a nutrient-deficient atmosphere, remove toxic substances, and act on recalcitrant compounds.

#### 5.1.4. Plant–Microbiome Engineering

Advanced biotechnological approaches, including computational biology and meta-omics, have considerably improved our understanding of plant microbiomes and their interactions and facilitate the field application of PGP microbes [45]. Plant–microbiome engineering (integrated with synthetic biology) is increasingly recognized as a feasible approach to confer PGP advantages to the host plant [226,228]. The precise and targeted microbiome engineering allows microbial selection, based on their functions (PGP traits), and may be delivered to specific plant species and plant parts at different developmental stages. In the top-down engineering approach, the desired traits are introduced in situ into different hosts via horizontal gene transfer (HGT). A possible top-down strategy includes the use of mobile genetic elements (MGEs) to transfer exogenous genes into random microbiomes. In the bottom-up engineering approach, first, microbes are isolated from the plant species [229] and engineered for specific traits and assembled as synthetic communities (SynComs) [230]. These approaches facilitate the integration of PGP genes and pathways into targeted plant beneficial microbes and stable maintenance of the PGP trait through genome-level engineering.

Currently, microbiome engineering–mediated modulation of the plant holobiont has emerged as a powerful technique to enhance plant stress resilience and crop yields [231]. Current advances in next-generation sequencing have facilitated understanding of the microbiome’s impact on plant performance [232]. Plant roots release a complex mixture of organic substances (including amino acids, secondary metabolites, organic acids, and sugars) that determines the functional attributes of the rhizosphere microbiome. These exudates, secreted from plant roots, serve as nutrients for microbes, affecting microbial diversity and growth [232,233], signaling plant-microbial communication [234,235], and deterring plant pathogens [236]. Moreover, environmental changes and stages of plant growth affect the amount and composition of root exudates, thereby aiding plant adaptation to pathogen infection, changes in nutrient availability, and drought conditions [237].

The current advances in agriculture highlight the prospects of precise microbiome engineering, microbiome breeding, and transplantation, offering a sustainable and scalable alternative to conventional procedures. The emerging prospects of rhizosphere microbiome engineering are immense, with potential to restore soil health, adapt to stressful conditions, and foster plant growth for sustainable outcomes [233]. Particularly, the competition between bacterial species in the rhizosphere zone is a critical determinant of the fitness of beneficial microbes and nitrogen-fixing rhizobia [238,239]. Engineering plant–microbiome associations through advanced biotechnologies and optimizing root exudates highlights a promising approach to boost crop productivity. Synthetic microbial communities mimic natural microbial populations and serve as promising tools for elucidating plant–microbial dynamics, particularly for understanding stress responses and nutrient acquisition mechanisms. Furthermore, studies on synthetic microbial communities have shown that differences in microbial species and nutritional conditions influence the pattern of microbial colonization, suggesting that plant immune signaling governs microbial composition [240].

**Figure 3:**
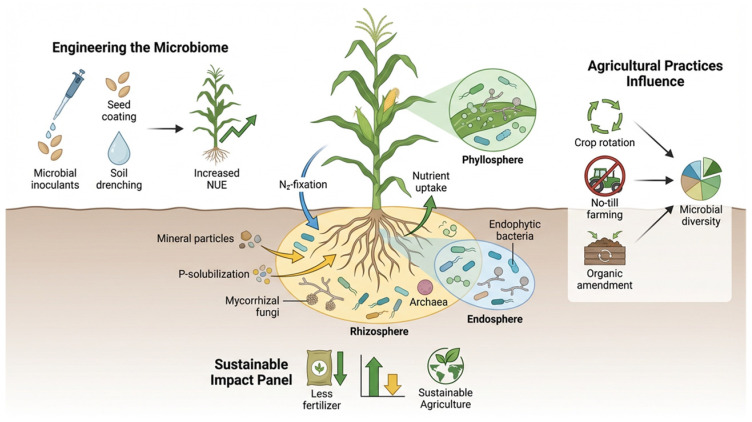
Harnessing plant microbiomes to promote Nutrient Use Efficiency (NUE) in agricultural crops. Plant–microbiome engineering defines a powerful tool to increase NUE and have a sustainable impact (less fertilizer usage) on agricultural practices (source: created with FigureLabs for scientific illustrations).

To promote plant growth and stress tolerance, the rhizosphere microbiome can be harnessed in multiple ways. For example, the natural microbial population can be transferred to establish a sound mutualistic association and promote microbial functionalities. In key crops like *O. sativa* and *Z. mays*, specific bacterial taxa facilitate the development of robust microbiomes [239,241]. Subsequently, microbial engineering approaches focus on enhancing plant-microbial symbiosis, integrating microbes into existing communities, and identifying core microbiomes to understand functional implications [242,243,244]. While the prospects of engineering microbiomes for targeted outcomes are promising, success depends on understanding intricate plant–microbial dynamics. Advanced biotechnological tools, including transcriptomics, CRISPR-based methods, and metagenomics, are instrumental in decoding these interactions [233]. Plant genes that affect microbiome functions and root exudate synthesis can be edited using CRISPR tools, improving plant performance [245,246,247]. Once the synthetic microbial communities are established, plant root exudates positively impact microbial functions: the growth and activities of these species are enhanced by nutrients supplied from root exudates [248]. Moreover, the activity of synthetic microbial communities in nutrient cycling (phosphate solubilization and nitrogen fixation) is promoted by plant root exudates [51]. Studies have shown that root exudates induce gene expression involved in nitrogenase synthesis in nitrogen-fixing bacteria [215,249]. The creation of tailored microbial communities is particularly significant for improving overall plant performance, stress tolerance, and nutrient cycling [250,251]. It also highlights a promising approach to improve plant health and productivity by targeting microbial functions and precisely tuning microbial interactions [52]. Figure 3, Harnessing plant microbiomes to promote Nutrient Use Efficiency (NUE) in agricultural crops.

## 6. Conclusions

Global agriculture is significantly impacted by rapid climate change, highlighting an urgent need to develop climate-resilient crops with high nutritional value and achieve global food security [252]. An integral aspect of sustainable farming comprises efficient nutrient utilization by crops (N and P in particular), attributed to its crucial influence on ecological balance and agricultural productivity. However, in recent times, rapid changes in the climate have intensified the complexities of nutrient management, with changes in precipitation patterns, higher global temperatures, and abrupt weather fluctuations, adversely impacting crop nutrient acquisition and utilization efficiencies. The global statistics suggest that major crops, including rice, wheat, and maize, rely heavily on fertilizers, projecting an urgent requirement for sustainable bio-based solutions to minimize adverse environmental impact.

To gain a deeper understanding and optimize nutrient utilization, it is important to study processes involved in nutrient acquisition, including plant-microbe associations, root functions, nutrient uptake and transport, plant growth promoters, and microbial communities. Subsequently, it is equally important to devise effective nutrient management strategies for yield improvement and climate-resilient agriculture. While traditional approaches in fertilizer management and biostimulant applications have achieved considerable success, biotechnological advances are significant in engineering crops for NUE traits, with major success achieved in improving plant nutrient uptake and utilization efficiency, stress tolerance, and improved soil-binding properties through root architecture modifications.

Plant-associated microbiomes are key players that promote plant growth and productivity, prominently impacting global agriculture [253]. The plant-microbial symbiosis, particularly AMF and fungal endophytes, promotes plant growth, affects water and nutrient uptake, while deriving nutrition from the plant’s photosynthesis, demonstrating a symbiotic alliance. Plant–microbiome engineering has been a major area of investigation in present times, aimed at harnessing the inherent functions and diversity of plant and soil-inhabiting microbial communities. Plant breeding and management approaches (for root exudates manipulation) optimize the plant microbiome for improved functions through selectively recruiting beneficial microbes. While tailoring microbial consortia according to specific crops and environmental conditions defines key potential, bridging knowledge gaps, including the factors impacting microbial colonization, plant–microbiome dynamics, and adopting precise editing approaches, are crucial to boost sustainable outcomes and crop productivity.

## Data Availability

No new data were created or analyzed in this study. Data sharing does not apply to this article.
